# Investigation of the Mechanical Properties of Miura-Ori Auxetic Woven Fabrics with Variable Initial Dihedral Fold Angles

**DOI:** 10.3390/ma18245663

**Published:** 2025-12-17

**Authors:** Qiaoli Xu, Yuan Tian, Zhaoqun Du

**Affiliations:** 1 College of Textiles and Fashion, Intelligent Textile Institute of Innovation, Hunan Institute of Engineering, Xiangtan 411104, China; 2 College of Textiles, Donghua University, Shanghai 201620, Chinaduzq@dhu.edu.cn (Z.D.)

**Keywords:** auxetic materials, woven fabrics, Miura origami, mechanical properties

## Abstract

Auxetic textiles, characterized by a negative Poisson’s ratio, offer considerable promise for innovative applications across multiple fields. In our earlier work, Miura-ori-inspired auxetic fabrics with three different initial dihedral fold angles—30°, 45°, and 60°—were successfully fabricated via jacquard weaving. Their fundamental auxetic behaviors were evaluated, showing deformation characteristics consistent with those in their geometric models. This study further investigates the mechanical properties of Miura-ori-based auxetic woven fabrics. Tensile testing, air permeability measurement, compression performance assessment, and repeated-loading cyclic rope-stretching tests were performed on the three fabric variants. The results show that the fabrics exhibit excellent air permeability, which increases with the proportion of the folded areas; the highest air permeability was observed at Miura-30°. Moreover, Miura-60° exhibited superior compression resistance. The fabrics also demonstrated outstanding structural stability under cyclic tensile loading, exhibiting optimal elastic recovery at the 30° configuration. Collectively, these findings provide a solid theoretical basis for future applications of Miura-ori auxetic woven fabrics.

## 1. Introduction

Auxetic materials, characterized by their unique negative Poisson’s ratio (NPR) behavior, expand transversely when they are stretched and they contract when they are compressed. This counter-intuitive mechanical response endows them with enhanced properties such as superior shear resistance, indentation resistance, fracture toughness, and improved energy absorption capabilities compared to conventional materials [1,2]. These remarkable characteristics have spurred significant research interest across various fields, including aerospace, biomedical engineering [3], automotive industry [4], protective equipment [5,6], and smart textiles [2,7,8,9].

The development of auxetic textiles [10] represents a pivotal advancement in textile materials, offering the potential to integrate NPR behavior into flexible, drapeable, and wearable structures [3,11,12,13,14,15,16]. In recent years, the principles of origami, the ancient art of paper folding [17], have provided powerful inspiration for designing novel mechanical metamaterials [18,19,20,21], including those with auxeticity [22]. Origami structures offer an elegant pathway to program mechanical properties through their crease patterns and folding mechanics; thus, origami has been identified as a promising basis for creating auxetic materials. Its deformation mechanisms naturally exhibit negative Poisson’s ratios in the planes, making origami an ideal candidate for translation into textile structures [23,24].

Building upon this concept, a pioneering attempt was made to fabricate an auxetic fabric by integrating the Miura-ori pattern into a weft-backed weave structure, as reported in the early study. This work successfully demonstrated the feasibility of producing a textile with an engineered Miura-ori pattern and qualitatively confirmed its in-plane and out-of-plane deformations [25]. It marked a significant step forward in bridging the gap between origami engineering and textile science.

While the preliminary validation of geometric deformation is crucial, a further and full understanding of the fundamental mechanical properties is essential for any material targeted for practical use.

For textiles, properties such as tensile strength, compression resilience, and performance under cyclic loading are critical determinants of their durability, comfort, and functional efficacy. The aforementioned study did not include a systematic quantification of these essential mechanical performances. This knowledge gap limits the potential for engineers and designers to confidently consider this novel fabric for real-world applications, as its behavior under various mechanical loads remains largely unexplored.

Therefore, to bridge this critical gap, the present study aims to conduct a thorough mechanical characterization of the previously developed weft-backed weave fabrics based on the Miura-ori structure. We will systematically investigate its tensile and compressive properties, with particular attention being paid to its behavior under repeated tensile loading. This quantitative analysis will provide foundational data on the mechanical robustness and energy dissipation characteristics of the fabric. The findings from this work will significantly deepen our understanding of the structure–property relationships in origami-inspired auxetic textiles and provide a vital scientific basis for evaluating their potential in advanced applications such as adaptive wear, impact-protective gear, and deployable structures.

## 2. Materials and Methods

Through combinations of yarn and fabric structure design, fabrics with a Miura-ori structure were manufactured with initial dihedral angles of 30°, 45°, and 60°, respectively. The fabric weave structure design, weaving machine, and weaving process have been detailed in previous articles [25]. The designed warp density was 536 ends/10 cm, and the weft density was 900 picks/10 cm. After the fabrics were removed from the loom, the densities increased slightly in both directions due to weaving shrinkage. According to the designed drafting diagram presented in our earlier article, the proportions of the crease zones were 11.76%, 9.66%, and 6.80%, respectively. The measured off-loom grammage was 272 g/m^2^, 261 g/m^2^, and 252 g/m^2^, accordingly.

For origami-based structural fabrics produced by weaving, the most significant distinction from structures made of paper or plastic is their air permeability, which is a critical factor that must be considered in various applications, such as flexible wearable devices. In this study, air permeability tests were conducted using a YG461E Air Permeability Tester (Wenzhou Fangyuan Instrument Co., Ltd., Wenzhou, China). The tests were performed in accordance with the GB/T 5453:1997 [26] standard, using a sample area of 20 cm^2^. The tests were carried out under pressure differentials of 50 Pa, 100 Pa, 200 Pa, and 500 Pa. A series of tests, including manual tensile, torsional, and bending tests, were performed on the Miura-45° fabric to evaluate its deformation behavior.

**Figure 1:**
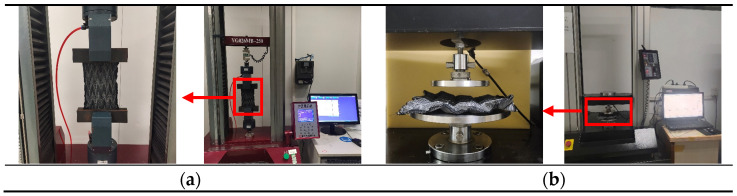
Tension and compression test of the Miura-ori auxetic woven fabrics: (**a**) tension test; (**b**) compression test. The red boxes and arrows in the left panel correspond to the magnified areas.

Tensile properties represent one of the most fundamental mechanical characteristics of textiles. To evaluate the tensile performance of the Miura-ori auxetic jacquard fabrics, an YG026MB-250 Multi-function Electronic Fabric Strength Tester (Shenzhen Fangyuan Instrument Co., Ltd., Shenzhen, China) was employed to conduct tensile recovery tests, seen in Figure 1a. The tests were performed in accordance with the Chinese standard FZ/T 70006 [27], using a constant elongation method. Specific parameters included a pre-tension of 0.3 N, an extension speed of 100 mm/min, a load dwell time of 1 s, and a recovery speed of 100 mm/min.

The three-dimensional configuration of the Miura-ori structure holds significant potential for applications in compression energy absorption. To evaluate its cushioning performance, compression tests were conducted using an HD026G Compression Tester (Nantong Hongda Experimental Instrument Co., Ltd., Nantong, China), equipped with a force sensor with a range of 200 N, as seen in Figure 1b. A flat platen test (with a cross-sectional diameter of 20 cm) was conducted under constant-compression-rate conditions. A pre-tension of 0.2 N was applied, with both compression and recovery speeds set to 20 mm/min. The initial height was set at 25 mm, and the specimen thickness was obtained through testing.

**Figure 2:**
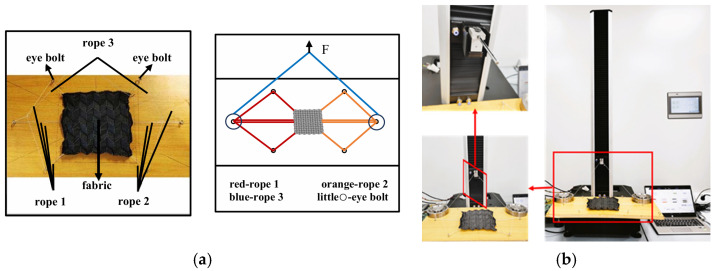
Rope stretching method and test of the Miura-ori auxetic woven fabrics: (**a**) rope stretching method; (**b**) rope stretching test. The red boxes and arrows in the left panel correspond to the magnified areas.

Due to the unique structural characteristics of the Miura-ori fabric, the conventional clamp-based fixing method for the stretching test was determined to be unsuitable and limited. Therefore, a custom-built rope-gripping fixture was employed to apply the tensile load. Figure 2 shows schematic and physical diagrams of the tensile test for the Miura-60° sample using a rope-clamping fixture. The experimental setup consists of three ropes, two smooth eye bolts, and a platform. Rope 1 and Rope 2 are of equal length and are fixed at three points along the fabric edge. The ropes can slide through the eye bolts with negligible friction. Rope 3 is used to connect Ropes 1 and 2 from the outside of the edge eye bolts. This configuration ensures that the load is uniformly distributed along the ropes, resulting in equal forces being applied to both sides of the origami structure. A self-made tension load cell was attached to the instrument to measure the force in Rope 3, in which the instrument is an HD026S Single-arm Multi-function Electronic Fabric Strength Tester, as seen in Figure 2b. In the test of this setup, the Miura-ori fabric deploys uniformly within the plane and can prevent the arching phenomenon.

**Figure 3:**
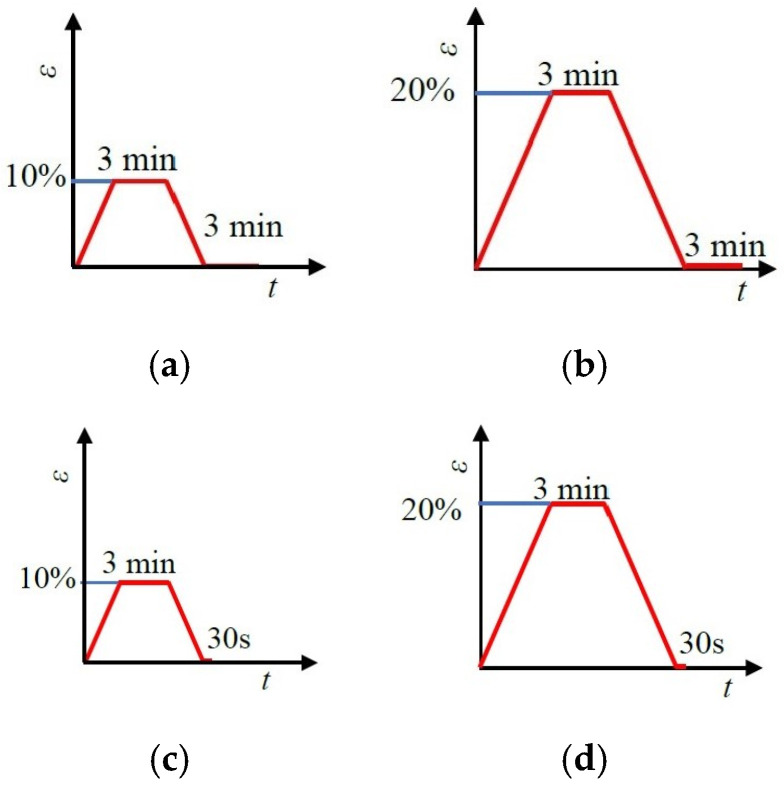
Elastic properties testing diagram for Miura-ori auxetic woven fabrics: (**a**) slow elastic deformation at 10% strain; (**b**) slow elastic deformation at 20% strain; (**c**) instantaneous elastic deformation at 10% strain; (**d**) instantaneous elastic deformation at 20% strain.

Elasticity is an essential property for the long-term use of auxetic textiles and a key indicator of fabric structural stability. For fabrics with an origami structure, the presence of creases makes structural stability even more critical. The elastic deformation capabilities of the Miura-ori fabrics were evaluated in this study by measuring both the immediate elastic recovery rate and the delayed elastic recovery rate, along with the plastic deformation rate. According to ISO 20932-1:2018 [28] (Textiles—determination of the elasticity of fabrics), in the immediate elastic recovery test, the fabric was stretched at a speed of 500 mm/min. To account for potential variations across different fabrics and strain levels, two strain levels—10% and 20%—were applied. The specific single-cycle testing procedure is illustrated in Figure 3.

**Figure 4:**
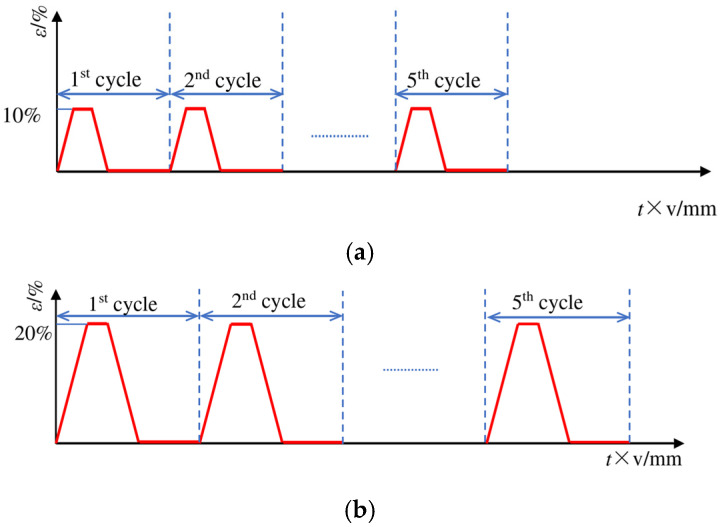
Curves of Miura-ori auxetic woven fabrics during cyclic tests: (**a**) 10% strain; (**b**) 20% strain.

Structural stability is critical for fabrics in daily use. To evaluate the deformation stability and durability of the Miura-ori fabrics, cyclic tensile and elasticity tests were conducted. A schematic diagram of this specific loading protocol is presented in Figure 4.

As can be seen in Figure 4, the fabric samples were stretched at a rate of 50 mm/min. Upon reaching the specified strain, the strain was maintained for 1 min. The fabric was then returned to its initial position at the same speed, followed by a 3 min static recovery period. This cycle was repeated five times, consecutively.

## 3. Results

### 3.1. Air Permeability and Deformation Behavior

#### 3.1.1. Air Permeability

**Figure 5:**
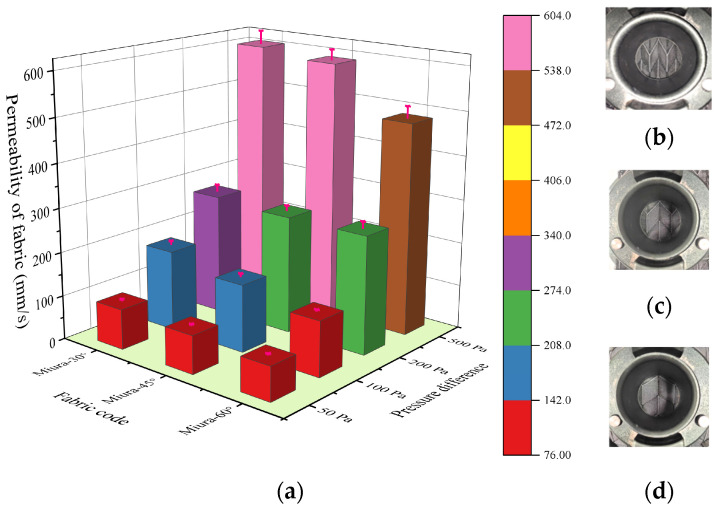
(**a**) Permeability of Miura-ori auxetic woven fabrics under different gas pressures. The physical images of (**b**) Miura-30°, (**c**) Miura-45°, and (**d**) Miura-60°.

Figure 5 shows the air permeability of the Miura-ori fabric under different pressure differences. The results indicate that as the pressure difference increases, the air permeability under different Miura-ori fabric configurations also increases. Moreover, as the angle increases, the air permeability shows a decreasing trend. Due to the tightly arranged yarns in the non-folded areas of the fabric and the presence of loose float yarns in the crease regions, the difference in air permeability may be related to the proportion of crease areas. Using SPSS Statistics (Version SPSS 27) for calculation, an analysis of the correlation between the proportion of crease areas and the air permeability was conducted, with the results presented in Table 1.

As clearly shown in Table 1, the covariances between all data sets are positive, indicating a consistent trend of change among the variables. The different variables are positively correlated. The Pearson correlation coefficients between all variables are greater than zero, consistent with the conclusion from the covariance, confirming a positive correlation. Furthermore, the Pearson correlation coefficients between the crease proportion and air permeability under different pressure differences are all greater than 0.95, except for the data under 200 Pa pressure, indicating a high correlation between the crease proportion and air permeability.

#### 3.1.2. Deformation Behavior

**Figure 6:**
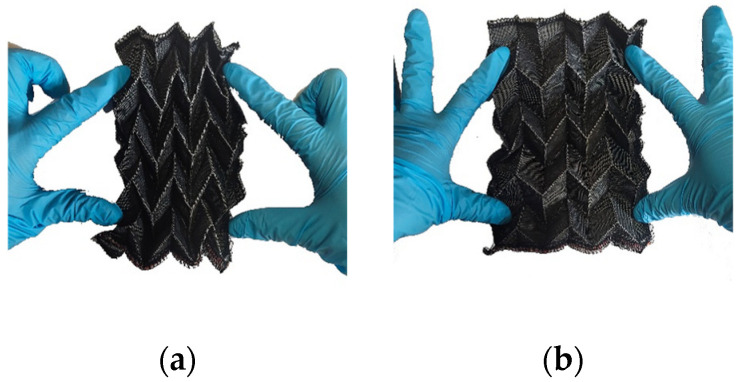

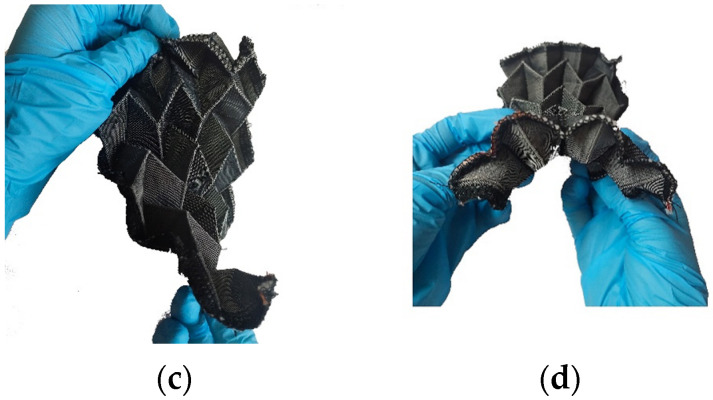
Mechanical behavior diagram of Miura-45°: (**a**) unstressed state; (**b**) tensile behavior; (**c**) torsion behavior; (**d**) flexural behavior.

As shown in Figure 6, during the stretching process, the Miura-ori-patterned fabric exhibits in-plane expansion behavior, which has a negative Poisson’s ratio effect in the plane; meanwhile, the dimension in the height direction decreases, representing a positive, out-of-plane Poisson’s ratio effect. The Miura-ori-patterned fabric is prone to twisting and does not form new creases during the twisting process. Additionally, when the Miura-ori-patterned fabric bends, it warps upward in the vertical direction, displaying a form similar to a hyperbolic paraboloid, resembling a saddle shape. This is referred to as the “saddle effect,” and this deformation morphology is related to the positive Poisson’s ratio exhibited by the Miura-ori out-of-plane structure.

#### 3.1.3. Stretching

As indicated in Table 2, the clamping length and stretched length differ across the tested specimens, which is attributable to the fact that the Miura-ori auxetic fabric features a pronounced three-dimensional geometrical configuration. When evaluated using the conventional flat-clamping mechanism of the YG026MB-250 tensile tester, the fabric’s transverse expansion is mechanically constrained. This restriction results in a notable structural response, thereby inducing a partial conversion of in-plane strain to out-of-plane displacement during extension. Consequently, the present analysis focuses exclusively on recovery behavior. The data presented in Table 2 reveal distinct recovery rates for the experimental elongations measured. Remarkably, under the flat-clamping condition, all three variants of the Miura-ori auxetic fabric exhibit exceptional tensile recovery performance, with the minimum recovery rate surpassing 78%.

#### 3.1.4. Compressing

Experimental data such as pressure (compression rate), compressive stress, compression work, and recovery work were recorded at a compression rate of 15%, because compressive deformation of textile fabrics typically remains within a relatively limited range. Moreover, excessive compressive deformation may lead to irreversible strain, resulting in poor recovery. Three tests were performed for each specimen.

As observed in Table 3, the initial dihedral fold angles significantly influence the energy absorption capacity of the Miura-ori auxetic fabrics. With an increase in initial dihedral fold angles from Miura-30° to Miura-45° and to Miura-60°, the fabric thickness, maximum pressure, compressive stress, compression work, and recovery work gradually increase, leading to a substantial enhancement in energy absorption capacity. Meanwhile, the coefficient of variation (CV) gradually decreases, which may be attributed to the fact that, among the three specimens, Miura-60° is the most stable, followed by Miura-45°, resulting in the smallest CV value for Miura-60°. Regarding the initial elastic stiffness (IES), Miura-45° exhibits the highest value.

**Figure 7:**
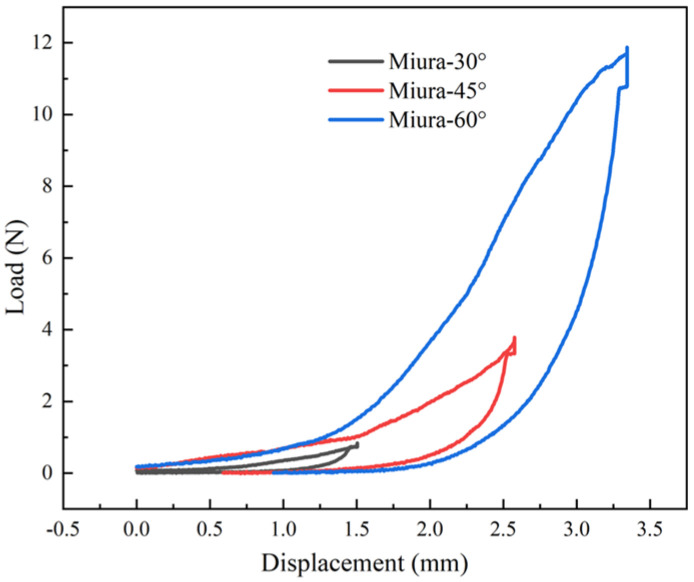
Relationship between compressive load and displacement of Miura-ori auxetic woven fabrics.

Figure 7 clearly shows that, as the initial dihedral fold angles increase, both the maximum pressure and the thickness increase (with the compression distance under a fixed 15% compression rate gradually increasing), accompanied by a significant improvement in the energy absorption capacity of the Miura-ori fabric: Miura-60° is the best.

### 3.2. Rope Stretching Under Repeated Load

#### 3.2.1. Elastic Recovery Rate

**Figure 8:**
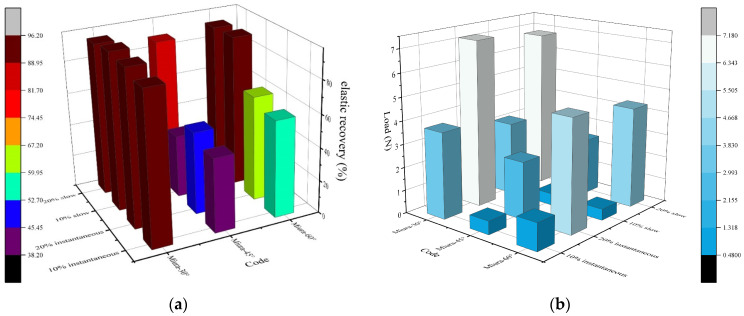
Elastic recovery rate of Miura-ori auxetic woven fabrics: (**a**) elastic recovery rate; (**b**) load.

To account for variations among different fabrics and to avoid discrepancies under different strain levels, two strain levels—10% and 20%—were applied. The single-cycle testing procedure is illustrated in Figure 8, and the results are presented in Table 4 and Figure 8.

As shown in Table 4 and Figure 8, the three specimens exhibit distinct elastic recovery behaviors. Miura-30° demonstrates the highest elastic recovery rate and the lowest plastic deformation rate. Both its immediate and delayed elastic recovery rates exceed 93% under 10% and 20% strain levels, indicating excellent elastic recovery performance. In contrast, Miura-45° shows the poorest elastic recovery, with certain cases of post-stretch recovery rates falling below 50%. Overall, Miura-45° exhibits substantial plastic deformation, which may be attributed to significant plastic deformation at the crease regions. Specifically, in Miura-45° specimens, the crease structure may have insufficient float length and width. As a result, when subjected to tensile deformation, it cannot recover rapidly like Miura-30°—which benefits from a high crease-area ratio and a large folding–expansion ratio at equilibrium—nor can it rely on long elastic float lines in the crease regions like Miura-60°, which has a smaller folding–expansion ratio at equilibrium. Therefore, while the recovery performance of Miura-60° is inferior to that of Miura-30°, it remains higher than that of Miura-45°.

Miura-45° fabrics may experience localized structural damage after stretching, preventing full self-recovery, which could be related to the structure’s self-stability. From Figure 8, it can be observed that Miura-30° exhibits the highest tensile force, corresponding to its superior elastic stability; meanwhile, Miura-45° shows the lowest tensile force and Miura-60° has an intermediate tensile force. The maximum force under 20% strain is consistently greater than that under 10% strain, which also helps explain the differences in elastic recovery rates among the three fabrics.

#### 3.2.2. Elastic Recovery

**Figure 9:**
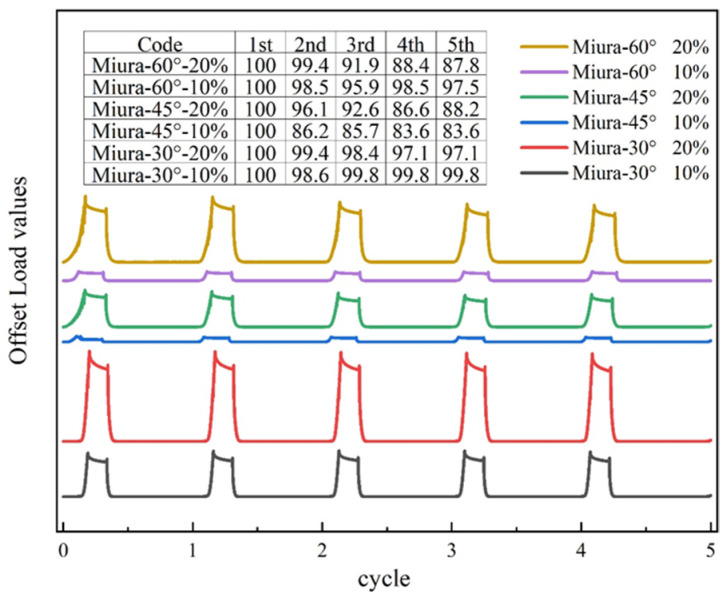
Cycle tensile test of Miura-ori auxetic woven fabrics.

As shown in Table 5 and Figure 9, under cyclic loading conditions, the elastic recovery rate and maximum tensile force of the fabrics show little difference compared to the results obtained from the immediate and delayed elastic tests at corresponding strain levels. The trend of variation among the different specimens remains consistent. Miura-30° exhibits the highest tensile force, followed by Miura-60°, while Miura-45° shows the lowest tensile force.

Figure 9 indicates a decrease in tensile force after five cycles. The curves stabilize after the second cycle, although a slight downward drift in maximum force is observed. After five cycles, the deformation capacity of all Miura-ori structures remains above 85%. Specifically, Miura-30° retains over 97% of its initial capacity, Miura-60° maintains above 87.8%, and Miura-45° shows the poorest shape retention, remaining just above 83.6%. The reasons for these differences have been analyzed in the previous section on immediate and delayed elastic recovery rates.

**Figure 10:**
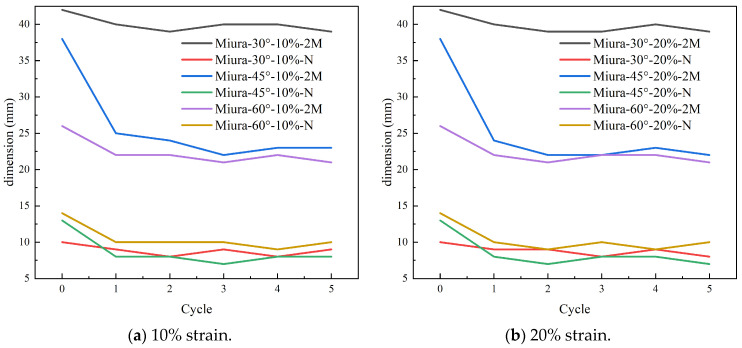
Dimension of Miura-ori auxetic woven fabrics in cycle tensile tests.

To capture the deformation behavior of the Miura-ori fabric during cyclic stretching, the dimensions of the fabric were measured after each cycle and before the commencement of the subsequent cycle. The resulting deformation data are presented in Figure 10.

As can be seen in Figure 10, upon the first tensile cycle, the Miura-ori-patterned fabric underwent substantial deformation, after which its response stabilized significantly in subsequent cycles. This phenomenon indicates excellent structural stability during stretching. Following the initial cycle, a notable reduction in fabric dimensions was observed, primarily attributed to the disruption of its pre-equilibrated state under constant temperature and humidity. Moreover, the dimensional changes showed little dependence on the applied strain levels of 10% and 20%.

## 4. Conclusions

Through mechanical performance testing of the prepared Miura-ori auxetic woven fabric, the following conclusions can be drawn:

1. The Miura-ori auxetic woven fabrics exhibit favorable air permeability, which is positively correlated with the proportion of creased regions: when the Miura-ori structure angle was 30°, air permeability was better.
2. According to the tensile and compressive properties of Miura-ori auxetic woven fabrics, Miura-45° showed the lowest recovery rate, which may be attributed to the minimal constraint on its elastic float yarns, which makes it difficult for the structure to recover.
3. The initial cycle induced considerable deformation in the Miura-ori auxetic woven fabric, with subsequent cycles exhibiting stabilized behavior, confirming its structural stability under tensile loading: when the Miura-ori structure angle was 30°, the elastic recovery was better.

## Figures and Tables

**Table 1:** Correlation analysis between the percentage of folding areas and the air permeability of Miura-ori auxetic woven fabrics.

Correlation Analysis		Crease Ratio	50 Pa	100 Pa	200 Pa	500 Pa
Crease Ratio	Pearson Correlation	1	0.996	0.995	0.771	0.951
	Significance (2-tailed)		0.058	0.062	0.439	0.200
	Covariance	6.199	17.600	71.139	8.281	155.488
50 Pa	Pearson Correlation		1	0.982	0.711	0.975
	Significance (2-tailed)			0.120	0.497	0.143
	Covariance		50.382	200.182	21.754	454.503
100 Pa	Pearson Correlation			1	0.830	0.916
	Significance (2-tailed)				0.377	0.263
	Covariance			824.344	102.754	1727.377
200 Pa	Pearson Correlation				1	0.537
	Significance (2-tailed)					0.639
	Covariance				18.588	152.010
500 Pa	Pearson Correlation					1
	Significance (2-tailed)					
	Covariance					4313.035

**Table 2:** Tension test results of Miura-ori auxetic woven fabrics.

Code	Miura-30°	Miura-45°	Miura-60°
Gauge Length/mm	150	130	100
Pre-set Elongation/mm	10	15	25
Measured Elongation/mm	1.79 ± 0.06 (3.39%)	3.51 ± 0.16 (4.69%)	1.21 ± 0.23 (19.08%)
Recovery Rate/%	84.70 ± 0.20 (0.24%)	78.37 ± 1.50 (1.92%)	95.20 ± 0.92 (0.96%)
Strain/%	1.00 ± 0.06 (2.56%)	2.50 ± 0.17 (6.93%)	1.23 ± 0.25 (20.40%)

**Table 3:** Compression test results of Miura-ori auxetic woven fabrics.

Code	Miura-30°	Miura-45°	Miura-60°
Fabric Thickness/mm	11.30 ± 0.43 (3.78%)	17.57 ± 0.42 (2.37%)	22.51 ± 0.08 (0.34%)
Maximum Stress/N	0.81 ± 0.13 (16.37%)	3.79 ± 0.03 (0.88%)	11.84 ± 0.21 (1.75%)
Pressure/Pa	26.00 ± 4.24 (16.32%)	121.00 ± 1.00 (0.83%)	377.00 ± 6.68 (1.77%)
Compression Work/mJ	0.71 ± 0.10 (14.80%)	3.29 ± 0.21 (6.44%)	13.97 ± 0.48 (3.43%)
Recovery Work/mJ	0.33 ± 0.04 (10.90%)	1.04 ± 0.10 (10.04%)	4.69 ± 0.23 (4.89%)
Recovery Rate/%	46.48	31.62	33.58
Linearity	0.52	0.33	0.35
Initial Stiffness IES/(N/m)	246.66	870.14	671.50

**Table 4:** Elastic recovery rate of Miura-ori auxetic woven fabrics.

Code	Classification	Strain/%	Elastic Recovery Rate/%	Plastic Deformation Rate/%	Maximum Tensile Force/N
Miura-30°	Instantaneous Elasticity	10	91.712	0.478	3.721
		20	94.939	0.399	7.176
	Delayed Elasticity	10	96.062	0.181	3.170
		20	93.365	0.503	6.809
Miura-45°	Instantaneous Elasticity	10	45.069	3.884	0.583
		20	50.265	8.254	2.486
	Delayed Elasticity	10	38.273	3.737	0.509
		20	88.351	0.933	2.424
Miura-60°	Instantaneous Elasticity	10	58.903	4.387	1.183
		20	63.921	6.756	4.829
	Delayed Elasticity	10	91.977	0.195	0.484
		20	91.197	1.015	4.320

**Table 5:** Cycle tensile test of Miura-ori auxetic woven fabrics.

Code	Strain/%	Elastic Response Rate/%	Plastic Strain Rate/%	Maximum Tensile Force/N
Miura-30°	10	95.567	0.209	3.324
	20	93.601	0.447	6.523
Miura-45°	10	43.903	2.172	0.427
	20	42.335	9.448	2.666
Miura-60°	10	69.832	1.265	0.682
	20	55.067	8.236	4.717

## Data Availability

The original contributions presented in this study are included in the article. Further inquiries can be directed to the corresponding author.
